# Heavy metal content of herbal health supplement products in Dubai – UAE: a cross-sectional study

**DOI:** 10.1186/s12906-019-2693-3

**Published:** 2019-10-21

**Authors:** Naseem Mohammed Abdulla, Balazs Adam, Iain Blair, Abderrahim Oulhaj

**Affiliations:** 1 Health and Safety Department, Dubai Municipality, Dubai, UAE; 2 Division of Occupational Health, Department of Preventive Medicine, Faculty of Public Health, University of Debrecen, Debrecen, Hungary; 3 Institute of Public Health, College of Medicine and Health Sciences, United Arab Emirates University, P.O. Box 17666, Al Ain, UAE

**Keywords:** Herbal supplements, Heavy metals, Limit of detection, Tolerable daily intake

## Abstract

**Background:**

Lead, mercury, cadmium, chromium, and arsenic intoxication have been associated with the use of health supplement (HS) products. The aim of this study is to estimate the concentration of heavy metals in HS products that are on sale in Dubai, United Arab Emirates, premises and to compare estimated daily metal intake with regulatory standards.

**Methods:**

Dubai-area premises selling HS products were identified by searching the Dubai Municipality database to identify all pharmacies, para-pharmacies and nutrition and healthcare shops. A total of 859 premises were identified in the Deira and Bur-Dubai areas. Data collection was performed between September 1 and December 12, 2016. During that period, all premises that had been identified within Dubai were visited and samples for laboratory testing were collected.

**Results:**

A total of 200 HS products were tested for lead, mercury, cadmium, chromium and arsenic. High proportion of samples were found to contain metals less than the limits of the detection (LOD) of the method. It was found that 93% of products contained Arsenic (As) < LOD, 94.5% of lead (Pb) < LOD, 100% of Cadmium (Cd) < LOD, 99% of Mercury (Hg) < LOD and 23.5% of Chromium (Cr) < LOD. Using the single imputation method to account for LOD, estimates for the average daily intake of lead was 0.88 μg compared to the tolerable daily intake (TDI) of 20 μg, daily intake of mercury was 0.09 μg (TDI = 20 μg), daily intake of cadmium was 0.83 μg (TDI = 6 μg) while for arsenic it was 0.92 μg compared to the tolerable daily intake of 10 μg. The average daily intake of chromium was 7.57 μg with no internationally established TDI. Assuming users followed the manufacturers’ instructions, daily intake of arsenic, lead and mercury would not exceed TDI for any of the 200 products. However, the daily intake of cadmium exceeded or approximated the TDI for three products.

**Conclusions:**

In this study we found low levels of metals in the products that were available for sale in Dubai. With few exceptions, if the products were used according to the suppliers’ instructions, average daily intake of heavy metals will be well below the recommended tolerable daily intakes.

## Background

The World Health Organization (WHO) and the United States Dietary Supplements Health and Education Act (DSHEA) of 1994 both define dietary supplements as products (other than tobacco) that are meant to supplement the diet. Their definitions include vitamins, minerals, herbs, botanical products and amino acids [1]. Dietary supplements are widely consumed and their availability in the global market has been increasing in recent years. They are readily available without prescription and their regulation is not as stringent as for medicines. Globally the supplement products are named or categorized as Dietary/Food/Health Supplements and controlled / monitored by authorities through their regulations. In USA these products are categorized as Dietary supplements and monitored by Food and Drug Administration (FDA), in Europe as food supplements and monitored by European Food Safety Authority (EFSA) and in Canada as Natural Health Products and monitored by Health Canada. The Consumer Products Safety Section (CPSS) at Dubai Municipality, UAE categorized these products as Health Supplements (HS) and defines as products, other than tobacco, anticipated to complement the diet including one or more of any dietary ingredients such as vitamins, minerals, herbs or other botanical, and/or amino acid ingredients [2] .The products with herbs and botanicals which is anticipated to complement diet fall under the category of Dietary/Food/Health Supplements, and as per CPSS definition, it is categorized as HS.

The use of health supplements (HS) is increasing worldwide and many consumers consider them as safe and more effective than conventional medicines. Ready availability without prescriptions and extensive advertising make HS a first choice for the treatment for many ailments. Supplements are preferred to conventional medicines for the treatment of digestive conditions and common respiratory ailments and for weight management [3]. In the United States (US), the use of HS is increasing year by year. Statistics show that 65% of the population in 2009, 66% in 2010 and 69% in 2011 were using health supplements [4]. The demand for HS is also increasing globally with a global market worth of $243 billion in 2014 [5]. Visits to providers of complementary and alternative medicine (CAM) exceed those to primary care physicians. In United States, Annual out-of-pocket cost of CAM (complementary and alternative medicine) is more than $30 billion with herbal HS products accounting for the major proportion of these treatments [6]. In the US, HS sales reached $28.1 billion in 2010, a 4.4% growth since 2009. The top ranked HS categories are: multivitamins ($4.9 billion), sports nutrition powders and formulas ($2.8 billion), B vitamins ($1.3 billion), calcium ($1.3 billion), and fish/animal oil ($1.1 billion) [3].

### HS use in Dubai and in the Middle East

Dubai in the United Arab Emirates (UAE) has a HS market that is expanding year by year. According to recent studies, the use of HS including herbal preparations has also increased in the Middle East [7]. The number of HS premises in Dubai has increased from 690 in 2014, to 740 in 2015, and 800 premises in 2016 [2]. The increasing number of online applications for importing health supplements provides further evidence for the growth of the HS market in Dubai. In addition, the number and total weight of HS consignments that have been imported through Dubai ports and inspected by the CPSS increased greatly between 2012 and 2015 (Table 1).

**Table 1:** Number and total weight of HS consignments in Dubai, 2012–2015 [2]

Year	Number	Gross Weight (kg)
2012	2019	2,940,877.89
2013	2448	3,790,542.40
2014	3224	4,702,010.22
2015	3752	4,790,351.00

### Contamination of health supplements

Unlike pharmaceuticals, in which the safety profile is well documented and closely monitored with established regulatory mechanisms, many consumers consider HS products as “harmless” and safe for consumption without necessarily undergoing thorough clinical testing. Even though established regulatory and monitoring policies are in place in many countries, adverse events caused by HS may not be reported adequately [8], Office of Inspector General, Department of Health and Human Services, USA [9]. One of the most serious concerns is with the consumption of herbal HS which are prone to contamination and adulteration. Adulteration of herbal products with undeclared pharmaceuticals and substitution with artificially manufactured substances have been reported [10]. In addition, contamination with dusts, pollens, molds and fungi have the potential to cause significant adverse effects [11]. A study in 2011in Hong Kong showed a serious and under-recognized problem of adulteration of Chinese herbal anti-diabetic products with undeclared pharmaceuticals, including both registered and banned drugs [10]. Herbal supplements may also be contaminated with pesticide residues due to excessive use of pesticides during the cultivation and lack of good agriculture practices. Organochlorine pesticide residues were found in a number of Chinese herbal plants cultivated in China and sold in Hong Kong [12].

A further serious safety issues associated with herbal supplement is heavy metal contamination including contamination with lead (Pb), cadmium (Cd) and mercury (Hg). Such heavy metal contamination can be caused by industrial contamination and agricultural contamination. Industrial contamination may happen during the transportation of the products or from the manufacturing of the product. The transport of the product in open bedded truck will exhaust pollutants into the ingredients, and the product processed in substandard factory conditions allowing more chance for contamination of heavy metals [13]. Agricultural contamination of heavy metals determined by the place where the plant cultivated. It is affected by atmospheric depositions of heavy metals which may happen by the heavy metal emission from petrol engine motor vehicles and leads to bioaccumulation of the heavy metals in the plant (as heavy metals could not travel more to the other parts of plant after absorption by root). Heavy metal contamination of soil and contaminated wastewater used for irrigation also plays a vital role in agricultural heavy metal contamination [14, 15]. Heavy metal contaminated HS may affect nutrition by displacing biologically useful metals, such as calcium and zinc, and can cause serious adverse health effects including carcinogenesis and reproductive effects [16, 17]. The purpose of the investigation described in this paper was to assess the concentration of heavy metals in herbal HS that are available for sale in retail outlets in Dubai, United Arab Emirates and to evaluate the associated health risk by comparing estimated per capita daily metal intake with regulatory standards. The motivation for assessing the concentration of heavy metals was that Dubai market includes products from all around the world with some products originating from countries with poor monitoring system for health supplements. Another reason was that despite some products are labeled as originating from countries with good monitoring system for health supplements, these products might be counterfeit of the true ones and hence their actual content in heavy metals could be higher than the claimed one.

### Health effects of heavy metals

Lead has been identified as a human carcinogenic risk factor as declared by the US Environmental Protection Agency (EPA). This heavy metal has the ability to distress the body organs. If the human body is exposed to lead for a long period of time, then this might lead to poor neurological performance, weakness, an increase in blood pressure and anemia. Severe brain and kidney damage and in worst case fatality could also happen when the human body is exposed to high doses of lead. High levels of exposure to lead can also induce miscarriage in pregnant women and spermatogenesis in men [18]. The EPA has also identified both mercuric chloride and methyl mercury as possible causes of human carcinogenicity. Areas affected by excessive exposure to mercuric chloride and methyl mercury are brain, kidneys as well as developing foetus for pregnant women. Exposure to high doses of these heavy metals also affect the nervous system including reluctance, irritability, muscle rigidity, visual and auditory dysfunction, and memory impairment. When the human body is exposed to high doses of metallic mercury vapor even for a short period of time, effects such as abdominal distress (nausea, vomiting), circulatory abnormalities, respiratory damage, ophthalmologic irritation and skin rashes can happen [18].

## Methods

Dubai-area premises that sell HS were identified by searching the Dubai Municipality database which contains information on all pharmacies, para-pharmacies and nutrition and healthcare shops in Dubai. A total of 859 premises were identified in this way and these were visited between 01 September and 12 December 2016. At each location, one package of each HS product intended for oral use was randomly selected, regardless of the country of manufacture, using an established sampling procedure in practice at Dubai Municipality. Only products with Arabic or English labels were included. Each item was given a unique reference number to prevent the collection of duplicate samples and permit tracking. For each sample, the following details were recorded: brand name, country of origin/manufacture, product name, item category, sub-category, bar code, batch number, dosage form, size/volume, recommended dosage per package, area and license number of the shop from where the product was obtained. When more than one premise carried the same product (identical name, manufacturer, formulation, size/volume, bar code), the example that had been obtained first was used for testing and the others were returned. If two products had the same name but were made by different manufacturers or had different formulations (for example tablets and capsules), they were considered to be different and both were submitted for testing.

### Preparation of specimens

The HS products were sent for analysis on the day of collection to a laboratory accredited by Dubai Accreditation Center. A 0.5 g portion of each product was mixed with 7 ml nitric acid (HNO_3_) in a sealed Teflon microwave digestion vessel. A two-step procedure was used for digestion. The vessels were heated to 200 °C for 25 min before being allowed to cool to below 50 °C. After cooling and venting, the solutions were diluted to a final volume of 20 ml with deionized water.

### Measurement of metal content

Determination of heavy metals was carried out using Inductively Coupled Plasma Optical Emission Spectrometry (ICP-OES). The metal concentrations were reported as microgram per gram (μg/g). The instrument’s limit of detection (LOD) was 5 μg/g for lead, 20 μg/g for mercury, 10 μg/g for arsenic, and 10 μg/g for cadmium. To assess reproducibility, any specimen that contained any of the metals at initial testing was subjected to repeat testing. In addition, ten samples in which no heavy metals had been detected were randomly selected for re-testing. Values for “tolerable daily intake” (TDI) were taken from the American Herbal Products Association guidance which is based on data from various national and international organizations [19]. TDI values used in the study are summarized in Table 2.

**Table 2:** Tolerable Daily Intake for selected metals [19]

Metal	μg/kg body weight	TDI for 70 kg for an adult
Arsenic (As) and its salts and derivatives	< 0.14	10.00 μg
Lead (Pb) and its salts and derivatives	< 0.29 μg/kg	20.00 μg
Cadmium (Cd) and its salts and derivatives	< 0.09 μg/kg	6.00 μg
Mercury (Hg) and its salts and derivatives	< 0.29	20.00 μg
Chromium (Cr) and its salts and derivatives	–	No internationally established TDI

The QC/QA procedures were carried out during the method development. For reagent blank, sample blank spike was prepared at each digestion level, and matrix spike was prepared at every 15 samples. For intermediate standard check, standard solution was run after the linearity as a verification of calibration curve using the same standard solution. Internal Quality Control standard (IQC) was prepared from different lot numbers and different concentrations which is not included in the linearity curve. A calibration verification solution was analysed after every 10 to 15 samples throughout the run. This is important for the system suitability measurement. The acceptance criteria was as follows: 1- Reagent blank should be less than the method detection limit. 2- The relative percent difference of duplicates and standards should be within 20%. 3- The percent recovery of sample blank spike and matrix spikes should be within 70 to 120% and 4- The percent recovery of intermediate check standard solution should be between 80 to 120%.

### Statistical analysis

For each product, an estimate of the daily intake of each metal was calculated as the product of the test concentration of the metal (μg/g) and the daily adult dose (g) of the product as advised by the manufacturer. The mean, median and standard deviation of the daily consumption of the five metals were computed. The average dose weight of the daily intake was not declared by the manufacturer. To deal with this, we calculated the average dose weight of the daily intake of herbal supplements by multiplying the weight of each capsule/tablet by the daily dose recommended by the manufacturing. The possible reasons for the variation in the average dose can be explained by variation of herbal supplement intake as recommended by the manufacturers’ instructions, different categories of herbal supplements, with different country of origins, different manufacturers, different batches and different dosage forms.

A considerable proportion of the measured concentrations of contaminating metals were below the LOD. One of the methods that can be used to account for these data is complete case analysis (CCA), where observations with values below the LOD are simply eliminated. This method is not appropriate in our study as many of these products probably have metal content under LOD and rejecting them would not be a preferred conservative estimation. Another method is single imputation, where every value below the LOD is replaced by a constant such as the mid-interval value, i.e. LOD/2, or the LOD itself. Single imputation using the upper bound of the interval (considered to be the most conservative method), single imputation using the middle point of the interval and the Kaplan Meir (KM) approach were used in this study to provide estimates of the mean, standard deviation and median for daily consumption of the five metals to allow comparison with the published TDIs. The KM approach is more accurate than single imputation. It is commonly used in survival analysis or time to event data when some observations are not completely observed and are then censored either to the right of to the left. This can be applied to our context as data under LOD can be considered as left censored. For more details about Kaplan Meir approach please see [20].

## Results

Samples from 200 HS products were analyzed. Of the 200 products, 137 (68%) were produced in the USA, 21 (10%) in Canada, 16 (8%) in the UK with the remaining 26 (13%) originating from other countries including Australia, UAE, Germany, India, New Zealand and Switzerland. Almost half of the sampled products were categorized as “herbal botanical”. Table 3 summarizes the number of test results where the measured concentration was below the LOD.

**Table 3:** Distribution of health supplement products that contained the tested metals below the limit of detection

	Number of products with metal content under LOD	
	n	%
Arsenic (As)	186	93.00
Lead (Pb)	189	94.50
Cadmium (Cd)	200	100.00
Mercury (Hg)	198	99.00
Chromium (Cr)	47	23.50

Estimates of the mean, standard deviation and median for daily consumption of the five metals using the three methods to account for LOD values are summarized in Table 4. The interpretation of this data is that these are the average daily intakes of the five metals amongst typical users of HS that have been purchased in Dubai. As discussed above, the single imputation (LOD) method gives the most conservative estimate of daily intake. Using this method, the estimates for the average daily intake of lead was 0.88 μg compared to the tolerable daily intake (TDI) of 20 μg, daily intake of mercury was 0.09 μg (TDI = 20 μg), daily intake of cadmium was 0.83 μg (TDI = 6 μg) while for arsenic it was 0.92 μg compared to the tolerable daily intake of 10 μg. The average daily intake of chromium was 7.57 μg with no internationally established TDI. It is reassuring that even using this estimate the average daily intake of metals are all well below the TDI. The Kaplan Meir (KM) method may give the most accurate estimates and it is notable that these are lower than those obtained with single imputation (LOD) and further below the TDI. The KM and LOD derived estimates for chromium are the most similar and this is a reflection of the fact that the test results for chromium had the smallest proportion of LOD values.

**Table 4:** Daily intake (μg) of five metals from health supplement products and tolerable daily intakes

	TDI	Single imputation (LOD)			Single imputation (LOD/2)			Kaplan Meir (KM)		
		Mean	Median	SD	Mean	Median	SD	Mean	Median	SD
Arsenic (As)	10.00 μg	0.92	0.65	1.12	0.53	0.33	0.81	0.45	NA	0.72
Lead (Pb)	20.00 μg	0.88	0.65	0.98	0.48	0.33	0.57	0.38	NA	0.37
Cadmium (Cd)	6.00 μg	0.83	0.64	0.96	0.41	0.32	0.48	NA	NA	NA
Mercury (Hg)	20.00 μg	0.09	0.06	0.13	0.04	0.03	0.06	0.01	NA	0.008
Chromium (Cr)	–	7.57	1.71	23.2	7.47	1.55	23.3	7.45	1.51	23.32

Estimates are made using three methods of accounting for measurements under the limit of detection

*NA* not available (missing), *LOD* limit of detection, *TDI* tolerable daily intake, *SD* standard deviation

**Fig. 1:**
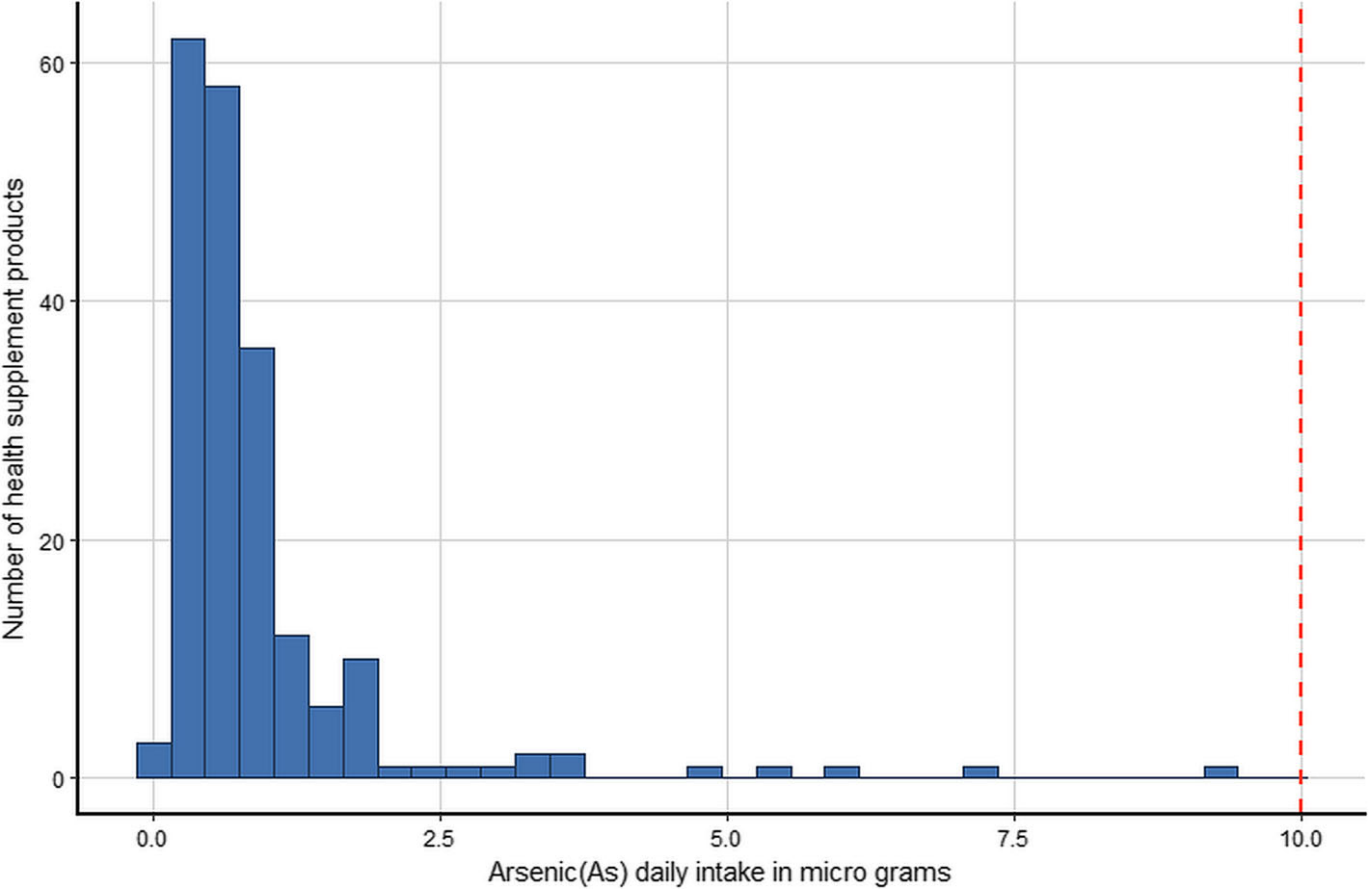
Histogram of the estimated daily intake (μg) of arsenic for health supplement products (*n* = 200). The vertical dashed line is the tolerable daily intake (TDI) of the metal according to heavy metal limit for Canada’s Natural Health products [19]

**Fig. 2:**
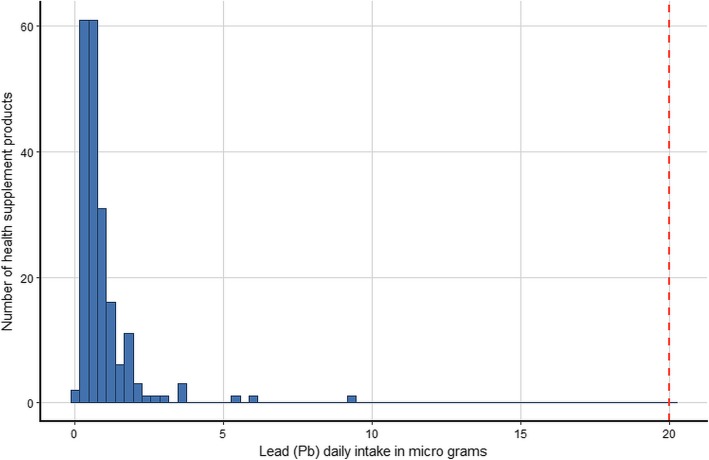
Histogram of the estimated daily intake (μg) of lead for health supplement products (*n* = 200). The vertical dashed line is the tolerable daily intake (TDI) of the metal according to heavy metal limit for Canada’s Natural Health products [19]

**Fig. 3:**
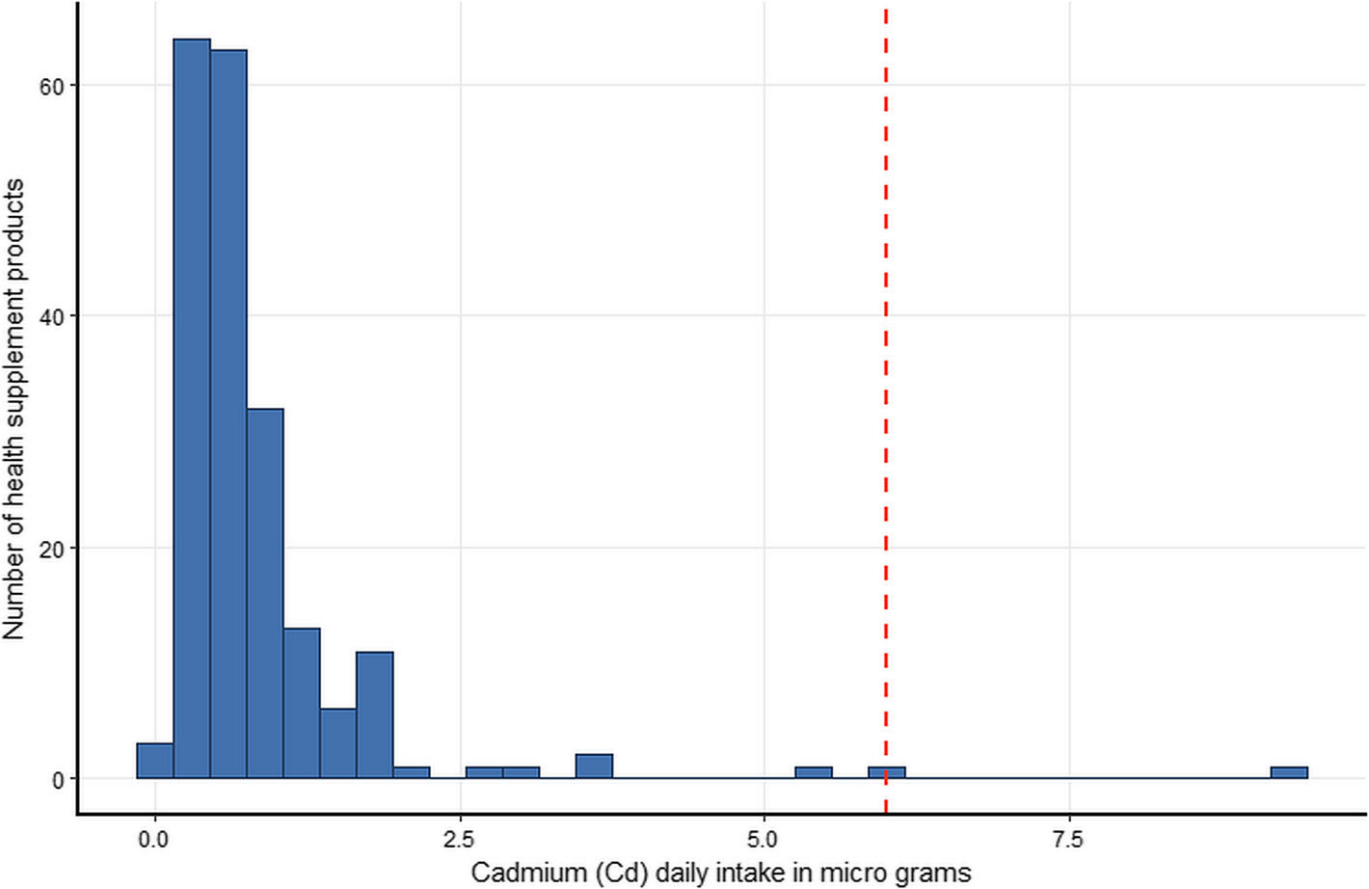
Histogram of the estimated daily intake (μg) of cadmium for health supplement products (*n* = 200). The vertical dashed line is the tolerable daily intake (TDI) of the metal according to heavy metal limit for Canada’s Natural Health products [19]

**Fig. 4:**
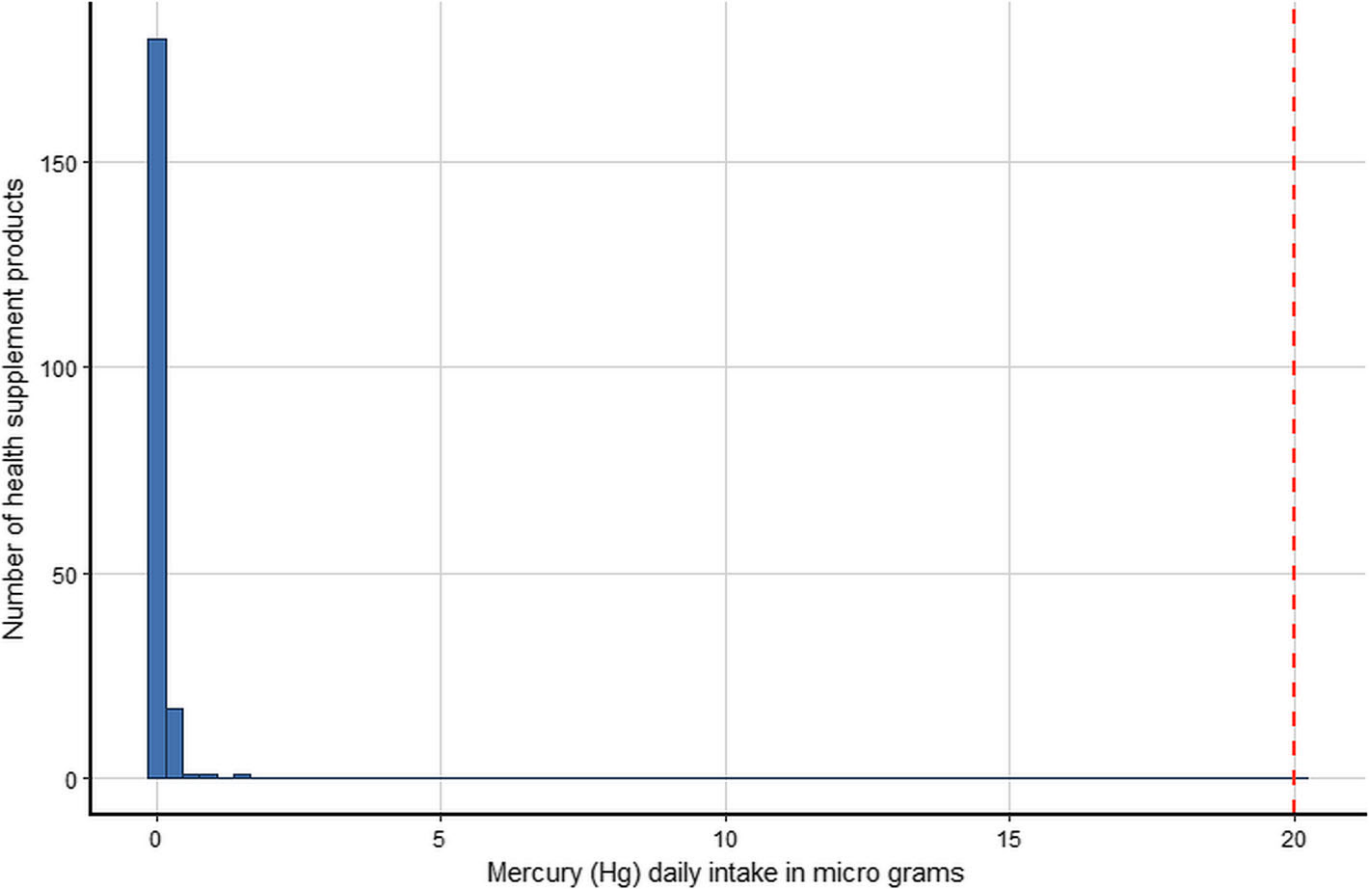
Histogram of the estimated daily intake (μg) of mercury for health supplement products (*n* = 200). The vertical dashed line is the tolerable daily intake (TDI) of the metal according to heavy metal limit for Canada’s Natural Health products [19]

**Fig. 5:**
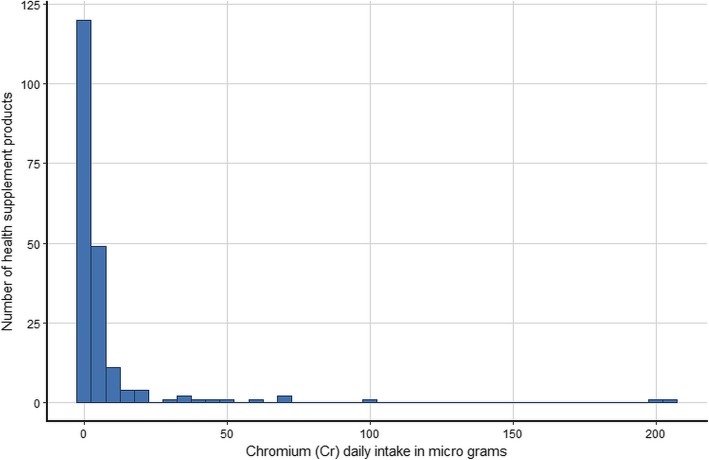
Histogram of the estimated daily intake (μg) of chromium for health supplement products (*n* = 200). The vertical dashed line is the tolerable daily intake (TDI) of the metal according to heavy metal limit for Canada’s Natural Health products [19]

The estimated daily intake of the five metals for the 200 products assuming that their use is in accordance with the manufacturers’ instructions and using the most conservative single imputation (LOD) method are shown graphically in histograms (Figs. 1, 2, 3, 4 and 5). The relevant TDIs are displayed as vertical “cut-off” limits.

None of the products exceeded the TDI for arsenic. The product with the highest daily intake (9.17 μg, LOD estimate) was of herbal origin, manufactured in the US. The product with the second highest daily intake (7.32 μg, actual value) was an herbal and mineral combination also of US origin. The product with the highest arsenic daily intake also gave the highest value for lead (9.17 μg, LOD estimate) although this was still below the TDI. Similarly, this same product had the highest daily intake for cadmium (9.17 μg, LOD estimate) which considerably exceeded the TDI (6.00 μg). The daily intake of two other products equaled or came close to the cadmium TDI, both were of US origin and contained herbal ingredients. The daily intake of mercury was low for all products and well below the TDI. There is no published TDI for chromium. Results show that some estimated daily intake values of chromium are considerably higher than those of the other examined metals, although for most products the dose is relatively low. Interestingly, the three products with the highest daily intake of chromium were not amongst the products that also had high intakes of the other metals, but they did cite “minerals” amongst their ingredients.

## Discussion

Previous studies have found heavy metal contamination of HS products and health effects have been reported. In one of the study conducted in Korea for Heavy metal toxicity of Traditional Chinese Medicines, 9 among the total 22 cases could directly attributed to the unacceptably high levels of heavy metals in these products. The common lead intoxication GI symptoms such as abdominal pain, anorexia, nausea, constipation, and anemia with basophilic stippling were also present in those patients [21].

Arsenic poisoning from herbal medicines has also been recorded. Arsenic poisoning from herbal medicine is usually chronic and presents with features, such as skin changes, leucopenia, anemia, sensory neuropathy, and malignancies [1]. The mercury toxicity may lead to the clinical consequences include hypertension, Coronary Heart Disease, Myocardial Infarction, generalized atherosclerosis, and renal dysfunction with proteinuria [22]. Through Cadmium toxicity, Cadmium may lead to concentrate in the kidney, particularly inducing proteinuria and renal dysfunction which will associated with hypertension [22].

However, in our survey of the heavy metal content of HS products available for sale in Dubai it is reassuring that very low levels were detected. In many cases the samples that were tested had metal concentrations that were below the LOD of the testing equipment. Using methods to account for LOD values, mean daily intake of metals were estimated based on adults taking the HS in the recommended dose and frequency. Estimates of mean daily consumption based on the most conservative method (single imputation LOD) were an order of magnitude below published TDI standards. When the same method was used to estimate the daily intake of the five metals from individual products, assuming that they were used according to the manufacturers’ instructions, similarly reassuring results were observed. Daily intake of arsenic, lead, and mercury did not exceed the TDI for any of the products. Although there is no published TDI for chromium, its mean daily intake was especially high for three products.

Our findings of low levels of the majority of heavy metals in HS available for sale and consumption in Dubai is generally satisfactory and reassuring. It may, in part, be due to the very close regulation of the HS market in Dubai by the regulatory authorities. While there are no reported instances of heavy metal toxicity from HS consumption in Dubai, awareness and vigilance amongst health professionals should be maintained and further improved. It might be noted that the increasing HS consumption among the Dubai population also significantly increases the potential risks that might be related to HS. The role of CPSS at Dubai Municipality in promoting the safe use of HS through import regulations and field inspections. Apart from the HS import data at CPSS, no studies exist till date to establish the extent of HS consumption in Dubai and the occurrence of adverse events, if any, that might be caused by their use. This warrants the need of an extensive study to assess the wide spread use of HS and to educate the health professionals and consumers about their safe use. The information collected could be of high significance and could also benefit in the realization of an ADR reporting system for HS, which in turn could promote the safe use of HS in Dubai.

Further testing of those products that had the highest estimates of metal intake is indicated, ideally using a more sensitive methodology with a lower LOD.

The strength of our study is that it is the first to sample and measure the heavy metal content of HS products in Dubai. The sampling method and sample size should ensure the representativeness and precision of our estimates. The analytical methods conformed to best practice and appropriate statistical methods were used to correct for LODs. These factors should improve the usefulness of our results.

Our study is not without limitations. The high proportion of measurements that were below LOD mean that inevitably our results are estimates which have levels of uncertainty attached to them. To overcome this limitation a number of different statistical methods have been used. Our conclusions are based on the method that gives the most conservative estimates. In addition, as was mentioned in the introduction and procedure, our study involved only finished herbal supplement products and excluded other forms of herbal supplements such as raw herbs.

## Conclusions

It is known that heavy metals can contaminate HS products particularly those derived from plants and toxic health effect have been reported. In our study we found low levels of metals in the products that were available for sale in Dubai. With few exceptions, if the products were used according to the suppliers’ instructions, average daily intake of heavy metals will be well below the recommended tolerable daily intakes.

## Data Availability

Data can be obtained upon request.

## Abbreviations

ADR: Adverse Drug Reaction
As: Arsenic
CAM: Complementary and Alternative Medicine
CCA: Complete Case Analysis
Cd: Cadmium
CPSS: Consumer Products Safety Section
Cr: Chromium
DSHEA: Dietary Supplements Health and Education Act
EFSA: European Food Safety Authority
EPA: Environmental Protection Agency
FDA: Food and Drug Administration
Hg: Mercury
HS: Health Supplements
KM: Kaplan Meir
LOD: Limit of Detection
Pb: Lead
SD: Standard Deviation
TDI: Tolerable Daily Intake
UAE: United Arab Emirates
US: United States
WHO: World Health Organization
